# Functional characterization of *Plasmodium vivax* hexose transporter 1

**DOI:** 10.3389/fcimb.2023.1321240

**Published:** 2024-01-12

**Authors:** Jeong Yeon Won, Ernest Mazigo, Seok Ho Cha, Jin-Hee Han

**Affiliations:** ^1^Department of Parasitology and Tropical Medicine, School of Medicine, Inha University, Incheon, Republic of Korea; ^2^Department of Medical Environmental Biology and Tropical Medicine, School of Medicine, Kangwon National University, Chuncheon, Republic of Korea

**Keywords:** *Plasmodium vivax*, hexose transporter 1, *Xenopus laevis* oocyte, glucose, uptake, malaria

## Abstract

*Plasmodium vivax* is the most widely distributed human malaria parasite. The eradication of vivax malaria remains challenging due to transmission of drug-resistant parasite and dormant liver form. Consequently, anti-malarial drugs with novel mechanisms of action are urgently demanded. Glucose uptake blocking strategy is suggested as a novel mode of action that leads to selective starvation in various species of malaria parasites. The role of hexose transporter 1 in *Plasmodium* species is glucose uptake, and its blocking strategies proved to successfully induce selective starvation. However, there is limited information on the glucose uptake properties via *P. vivax* hexose transporter 1 (PvHT1). Thus, we focused on the PvHT1 to precisely identify its properties of glucose uptake. The PvHT1 North Korean strain (PvHT1_NK_) expressed *Xenopus laevis* oocytes mediating the transport of [^3^H] deoxy-D-glucose (ddGlu) in an expression and incubation time-dependent manner without sodium dependency. Moreover, the PvHT1_NK_ showed no exchange mode of glucose in efflux experiments and concentration-dependent results showed saturable kinetics following the Michaelis-Menten equation. Non-linear regression analysis revealed a Km value of 294.1 μM and a Vmax value of 1,060 pmol/oocyte/hr, and inhibition experiments showed a strong inhibitory effect by glucose, mannose, and ddGlu. Additionally, weak inhibition was observed with fructose and galactose. Comparison of amino acid sequence and tertiary structure between *P. falciparum* and *P. vivax* HT1 revealed a completely conserved residue in glucose binding pocket. This result supported that the glucose uptake properties are similar to *P. falciparum*, and PfHT1 inhibitor (compound 3361) works in *P. vivax*. These findings provide properties of glucose uptake via PvHT1_NK_ for carbohydrate metabolism and support the approaches to vivax malaria drug development strategy targeting the PvHT1 for starving of the parasite.

## Introduction

Malaria is one of the most prevalent infectious diseases caused by *Plasmodium* species. Estimates show that 247 million cases and 619 thousand malaria deaths occurred in 2021 (World Health Organization, 2022). Among the *Plasmodium* species, *P. vivax* is the most widely distributed worldwide with estimated 6.9 million cases in 2021 (World Health Organization, 2022). The number of *P. vivax* infections has gradually been increased since the onset of the COVID-19 pandemic (World Health Organization, 2022). This situation highlights the continued urgency required to eliminate this disease including early diagnosis, effective treatment, and prevention. Despite intense efforts in malaria control, there is still an emerging problem in treatment. To date, more than twenty antimalarial drugs have been approved (Haldar et al., 2018). However, drug-resistant vivax malaria has widely spread and poses a severe threat to global malaria control (Dayananda et al., 2018). Thus, there is an urgent demand for new antimalarial agents with novel mechanisms of action to achieve successful malaria control.

The asexual stage of *P. falciparum* growth and replication depends on a continuous supply of host-derived glucose (Kirk et al., 1996). The malaria parasite consumes 100-fold higher glucose than the host cell as the primary carbon source (Roth, 1990; Kirk et al., 1996). In previous studies, the lack of D-glucose or D-fructose effectively inhibited the growth of *P. falciparum in vitro* culture (Joet et al., 2003a; Slavic et al., 2010). The functional characteristics of *P. falciparum* hexose transporter 1 (PfHT1) has been intensively studied as a glucose transporter. PfHT1 actively uptakes host-derived glucose, and its analog compound 3361 (C3361) has shown growth inhibition activity (Jiang et al., 2020).

PfHT1 belongs to the major facilitator superfamily (MFS) and shares comparatively low sequence similarity with human glucose transporter 1 (hGLUT1), which is the primary glucose transporter of the host cell (Madej et al., 2014). Further, the MFS members share the core structure of the twelve transmembrane segments (Quistgaard et al., 2016). Despite the low sequence similarity, there is a conserved function for glucose uptake, offering an opportunity for ‘parasite starving’ approaches without affecting the host cell. Moreover, the HT1 in simian malaria *P. knowlesi* and rodent malaria *P. yoelii* have been confirmed for D-glucose and D-fructose uptake activity (Joet et al., 2002). Similarly, the *P. vivax* HT1 functions and its inhibitor C3361 has been investigated for its efficacy in killing of short-term *in vitro* cultures of *P. vivax* isolates from patients (Joet et al., 2004). In view of this, the glucose blocking strategy presents a novel potential mechanism of action for anti-malarial drug development. This study has highlighted that targeting PvHT1 for glucose uptake inhibition could potentially be effective in clearing of *P. vivax* infections.

Collectively, the major role of HT1 in various *Plasmodium* species is to transport glucose. The blocking strategy of HT1 is potentially a treatment that could transcend species boundaries. However, there is a lack of precise information regarding the properties of glucose uptake via PvHT1. Therefore, our study focuses on the functional characteristics of PvHT1 to understand the precise properties of glucose uptake, using the *Xenopus laevis* heterologous expression system. Besides, we presented *in silico* computational simulations that confirmed glucose and C3361 inhibitor binding mode of PvHT1.

## Materials and methods

### Chemicals

Radiolabeled compounds, including [^3^H] deoxy-D-glucose (32.5 Ci/mmol), [^3^H] arginine (50.5 Ci/mmol), [^3^H] glycine (45.2 Ci/mmol), [^14^C] α-ketoglutaric acid (54.8 mCi/mmol), [^14^C] lactic acid (154.8 mCi/mmol), and [^14^C] pyruvic acid (7.64 mCi/mmol), were purchased from Perkin-Elmer Life Science Products (Boston, MA, USA). All other chemicals and reagents were obtained from commercial sources, ensuring they were of analytical grade.

### Ethical statement

Study procedures on collection of human blood sample and laboratory investigations were reviewed and approved by the Institutional Review Board (IRB) of Inha University (Approval No. 2020-04-004).

### Computational analysis

*Plasmodium* species HT1 sequences were obtained from two databases: The National Center for Biotechnology Information (NCBI) (http://www.ncbi.nlm.nih.gov/) and PlasmoDB (https://plasmodb.org/). The amino acid sequences were aligned using the Clustal W method in Lasergene v11 MegAlign (Madison, WI) and conserved residues for glucose binding pocket were confirmed based on PfHT1 as a reference sequence (*P. falciparum* 3D7 strain). A phylogenetic tree was generated by the maximum-likelihood (ML) method with a bootstrap test for 1,000 pseudo-replications to enhance robustness using MEGA 11 software (Tamura et al., 2021). The membrane topology was determined using the TMHMM server (https://services.healthtech.dtu.dk/services/TMHMM-2.0).

The tertiary structure of PvHT1 model was generated by homology-based prediction of the SWISS-MODEL (https://swissmodel.expasy.org) server (Biasini et al., 2014). The quality and potential errors of the generated PvHT1_NK_ model were assessed using Ramachandran plots (Lovell et al., 2003) and ERRAT (Colovos and Yeates, 1993). To confirm the superimposition of PfHT1 (PDB ID: 6m20.1) and PvHT1_NK_ model, the root-mean-square deviation (RMSD) and Z-score were measured by DALI server (http://ekhidna2.biocenter.helsinki.fi/dali) (Holm, 2022). The RMSD values indicated structural differences between the aligned alpha-carbon positions and for a crystallographic model with approximately 50% sequence identity, the difference was around 1 Å. The Z-score measured the distance in standard deviations between the observed alignment RMSD and the mean RMSD for random pairs of the same length, with the same or fewer gaps. A Z-score lower than 2 indicated spurious similarities between two structures. Visualization of the structure was accomplished using UCSF CHIMERA software (Pettersen et al., 2004).

### *Plasmodium vivax* hexose transporter 1 (PvHT1) cloning

The total RNA was extracted from a vivax malaria patient whole blood, residing in Northern Gyeonggi-do. The Northern Gyeonggi-do area is near the Korean Demilitarized Zone (DMZ), which is known as a vivax malaria endemic area in South Korea. Total RNA was extracted from 200 μL of whole blood obtained from the patient. After centrifugation, serum was removed and blood cells were resuspended in an equivalent volume of phosphate-buffered saline. Subsequently, the resuspended cells were lysed using 750 μL of Tri reagent BD (Sigma, St. Louis, MO), supplemented with 20 μL of 5N acetic acid. The solution was incubated at room temperature for 5 minutes, and the total RNA isolation was performed according to manufacturer’s instructions. The RNA pellet was dissolved in 30 μL of nuclease-free water and stored at -70°C.

The first strand of cDNA was generated by reverse transcription using 5 µL of total RNA solution, 2 μL of 10 mM dNTP mix (ELPIS-Biotech, Daejeon, Republic of Korea), 200 units of M-MLV reverse transcriptase (Thermo Fisher, Waltham, MA), and 1 μL of 20 μM Oligo (dT) primer. The reaction was carried out at 42°C for 60 minutes. The prepared cDNA was used as the template for polymerase chain reaction (PCR) under the following conditions: 35 cycles at 94°C for 30 s, 58°C for 30 s, and 72°C for 90 s. Primers were synthesized containing *XhoI* or *BamHI* restriction enzyme sites (underlined): 5’- CTC GAG ATG AAG AAG AGC AGC-3’ (forward) and 5’- GGA TTC TCA CAC GGC CGA CTT GCC-3’ (reverse). The PCR amplicon was subsequently subcloned into the pGEM-T easy TA cloning vector (Promega, Madison, WI). For sequence confirmation of oligonucleotides, synthesized primers used a dye-termination method through the API Prism™ 3730 (Macrogen, Seoul, Republic of Korea). The PvHT1 sequence was verified to be an exact match with that of the *P. vivax* North Korea strain (PvHT1_NK_, NCBI accession number: KNA01348). Subsequently, PvHT1_NK_ was subcloned into the pBluescript II SK (+) vector for synthesis of complementary RNA (cRNA).

### cRNA synthesis and uptake experiment using *Xenopus laevis* oocytes

The plasmid DNA pBluescript II SK (+) was used for *in vitro* transcription. Capped cRNA was synthesized *in vitro* using mMESSAGE mMACHINE™ T7 transcription kit (Thermo Fisher Scientific) from the linearized plasmid DNAs with *XbaI*. Defolliculated oocytes were injected with 50 ng of capped cRNA and incubated in Barth’s solution (88 mM NaCl, 1 mM KCl, 0.33 mM Ca(NO_3_)_2_, 0.4 mM CaCl_2_, 0.8 mM MgSO_4_, 2.4 mM NaHCO_3_, 10 mM HEPES, and gentamicin 50 μg/mL) at a pH of 7.4 and temperature 18°C. After 2 days of incubation, uptake experiments were performed. Distilled water-injected oocytes (without cRNA)-(control) and PvHT1_NK_ cRNA-injected oocytes were incubated in 500 μL of ND96 solution (96 mM NaCl, 2 mM KCl, 1.8 mM CaCl_2_, 1 mM MgCl_2_, 5 mM HEPES, pH 7.4) containing radiolabeled substrates for 1 h. The transport experiment was terminated by the addition of ice-cold ND96, and the oocytes were washed 5 times with the same solution. Then, the oocytes were dissolved with 10% SDS and radioactivity was counted. For examining sodium dependency, the ND96 solution was substituted with 96 mM of lithium, choline, and N-methyl-d-glucamine (NMDG). To examine the trans-stimulatory effect on transport via PvHT1_NK_, oocytes expressing PvHT1_NK_ were preloaded with [^3^H] deoxy-D-glucose (300 nM in the medium) for 90 minutes. Subsequently, the washed oocytes were transferred into the medium with or without unlabeled deoxy-D-glucose or glucose (100 μM and 1,000 μM) for 60 minutes (Kusuhara et al., 1999). For the inhibition experiment, PvHT1_NK_-expressing oocytes were incubated for 60 minutes with various sugar substrates, ranging from 100 mM to 0.1 mM (10-fold diluted), along with 50 nM isotope-labeled deoxy-D-glucose and the indicated compounds.

### The kinetics of glucose uptake via PvHT1 _NK_ and statistical analysis

The kinetic parameters related to glucose uptake through PvHT1_NK_ were determined using the following equation: v = Vmax × S/(Km + S), where ‘v’ represents the rate of substrate uptake (pmol/hour/oocyte), ‘S’ stands for the substrate concentration in the medium (µM), ‘Km’ signifies the Michaelis-Menten constant (µM), and ‘Vmax’ denotes the maximum uptake rate (pmol/oocyte/hour). To ascertain these kinetic parameters, we applied the equation to the [^3^H] deoxy-D-glucose transport velocity, obtained by subtracting the transport rate in non-injected oocytes from that in PvHT1_NK_-expressing oocytes. This fitting process was carried out using an iterative non-linear least squares method with the MULTI program (Yamaoka et al., 1981). The input data were weighted based on the reciprocal of the observed values, and the Damping Gauss-Newton method was utilized for fitting. Subsequently, the fitted data were transformed into the 1/S versus 1/V format for a Lineweaver-Burk analysis.

## Results

### Schematic characteristics of *Plasmodium vivax* hexose transporter 1

**Figure 1:**
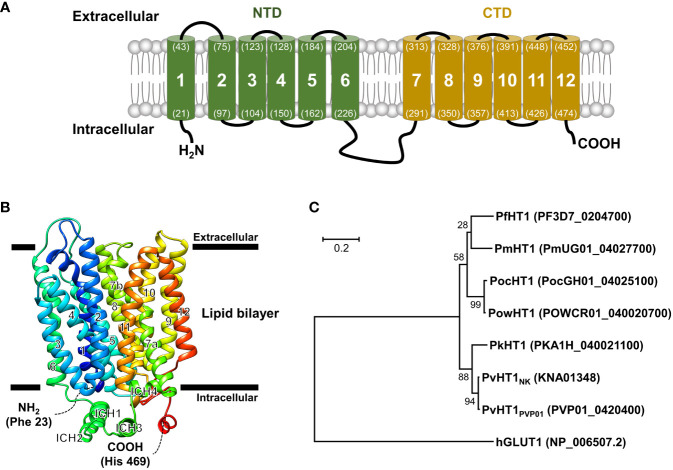
Schematic representation of the PvHT1_NK_. **(A)** The predicted topology of the full-length PvHT1 (1-502 aa.) showed 12 transmembrane segments with the intracellular facing short N-terminus (1-21 aa.) and C-terminus (478-502 aa.). The number of transmembrane domains and amino acid position is shown by parenthesis with residue number. **(B)** PvHT1 homology base model on *P. falciparum* hexose transporter 1 (PfHT1, PDB ID: 6m20.1) structure. The homology base model was constructed from Phe 23 (N-terminus, Blue color) to His 469 (C-terminus, Red color). The horizontal bars represent the expected lipid bilayer for the boundaries of the 12 transmembrane domains. **(C)** The phylogenetic tree of hexose transporter 1 in human malaria; *P. malariae* (Pm), *P. ovale* curtisi (Poc), *P. ovale* wallikeri (Pow), *P. knowlesi* (Pk), and human glucose transporter 1 (hGLUT1) (PlasmoDB or NCBI accession number shown in parenthesis) constructed using MEGA11 by ML method with 1,000 pseudo-replications showing the distance of relationship.

The PvHT1_NK_ gene is composed of 1,509 base pairs that encode a 502 amino acid with a calculated molecular mass of 55.8 kDa. The putative topological model of PvHT1_NK_ shares characteristic features and similarities with class I and II human glucose transporters (GLUT), including the 12 transmembrane domains (TMs) (**Figure 1A**). The typical characteristics of this class include type III multipass transmembrane proteins, which have intracellular N-terminus and C-terminus (**Figure 1A**). Additionally, TM6 and TM7 are connected by a long intracellular region that contains six transmembrane domains in both the N-terminal (NTD) and C-terminal (CTD) (**Figure 1A**).

The tertiary structure of PvHT1_NK_ was determined using the SWISS-MODEL server. The *P. falciparum* hexose transporter 1 (PDB ID: 6m20.1) was identified as a superior template, displaying the best-fitting structure. The quality assessment of the predicted PvHT1_NK_ model, evaluated via a Ramachandran plot, showed that 95.3% of the model occupied favorable regions, with 0.2% in disallowed regions. The preliminary ab initio model structure obtained a quality verification score of 97.2% based on ERRAT results in the final model (**Figure 1B**). The tertiary structure clearly showed the presence of 12 major alpha-helices within the lipid bilayer region in PvHT1_NK_. Additionally, a long loop exists in the intracellular region with four alpha-helices (ICH) that serves as a connecting bridge between TMs 6 and 7 (**Figures 1A, B**). Overall, the PvHT1_NK_ structure exhibited a conserved core structure shared with glucose transporters found in other organisms.

The relationship between the HT1 of human malaria parasite and human glucose transporter 1 (hGLUT1) (NCBI accession number: NP_006507.2) sequences was analyzed for similarity comparison. The hGLUT1 is a transporter for glucose uptake in host cells. The hGLUT1 gene showed comparatively low sequence similarity with *Plasmodium* species HT1 ranging from 22.6% to 23.4% (**Supplementary Figure 1**). In contrast, within malaria parasites, HT1 is comparatively well-conserved, showing more than 76.4% sequence similarity (**Supplementary Figure 1**). Consequently, the phylogenetic tree clearly distinguishes the significant distance between hGLUT1 and malaria parasite HT1 (**Figure 1C**).

### Comparison of hexose transporter 1 sequence in *Plasmodium* species

The *P. vivax* isolate was collected from North Gyeonggi-do near the DMZ in Korea and corresponds to the North Korea strain. Thus, we conducted an expanded analysis for the comparison of PvHT1 sequence of worldwide isolates. When compared with PvHT1_PVP01_ (PVP01_0420400) as a reference sequence, it showed 98.6% sequence similarity with PvHT1_NK_ (KNA01348), which was used in the present study (**Supplementary Figure 1**). The comparison of worldwide isolates for PvHT1 showed point mutations in the extacellular loop (between TM5 and TM6, and TM9 and TM10) within intra-species levels for two major phenotypes (**Supplementary Figure 2**). These natural sequence mutations were not detected in the putative glucose binding pocket. Similarly, in the case of HT1 of inter-species level, identical residues were found in critical glucose binding residues, with limited mutations observed in glucose binding pocket (**Supplementary Figure 3**).

### Uptake properties of PvHT1_NK_

**Figure 2:**
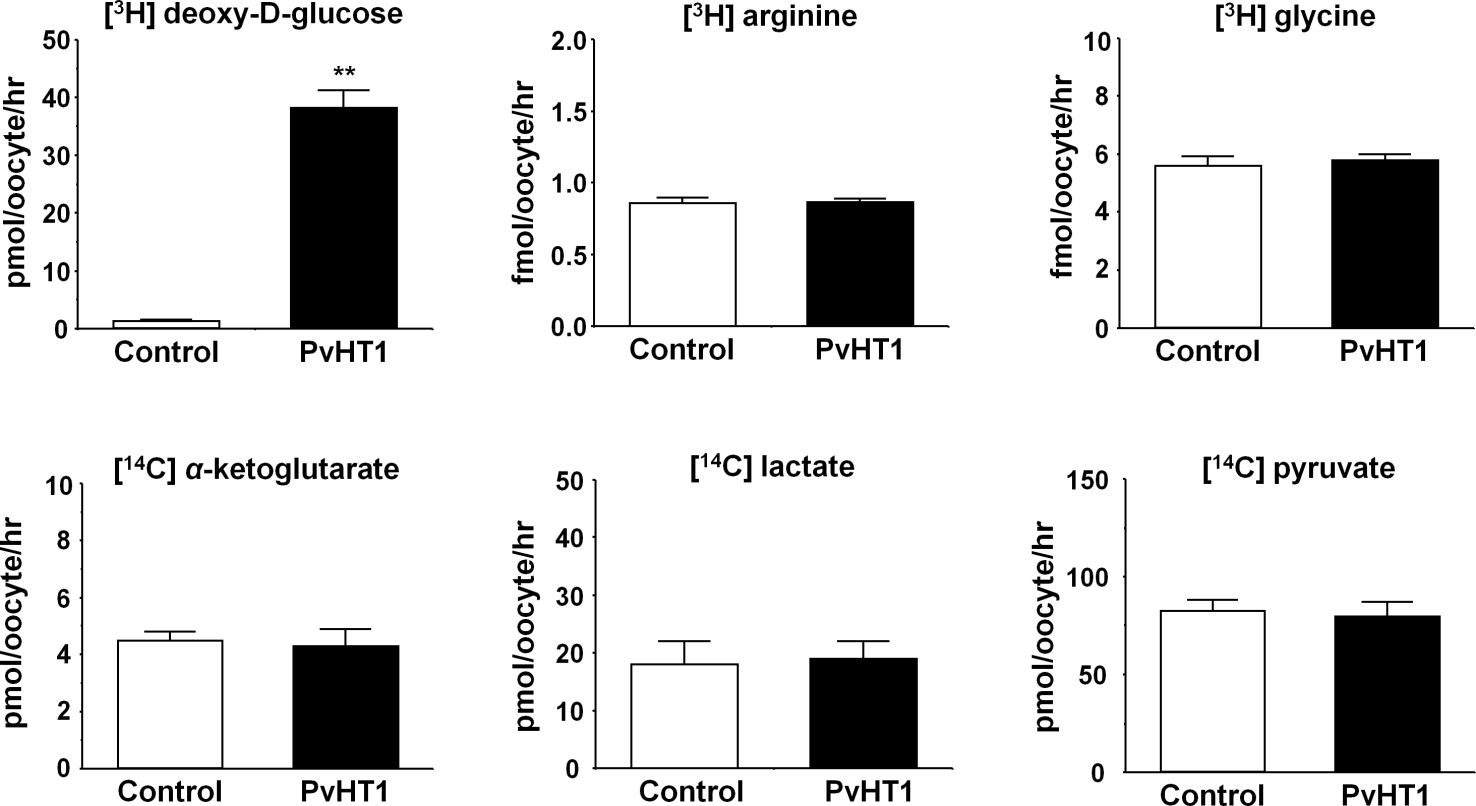
PvHT1_NK_ mediated uptake of D-glucose. The uptake rates of radiolabeled compounds were measured in water-injected (control, white bar) oocytes and PvHT1_NK_ (black bar) expressing oocytes for 1 hour (mean ± S.E., *n* = 8-10). The concentration of injected substrate as follows; [^3^H] deoxy-D-glucose, 300 nM; [^3^H] arginine, 100 nM; [^3^H] glycine, 100 nM; [^14^C] α-ketoglutarate, 5 μM; [^14^C] lactate, 1 μM; [^14^C] pyruvate, 1 μM. The student's-*t* Test (**p < 0.01).

The *Xenopus laevis* oocyte expression system was used to investigate the transport characteristics of PvHT1_NK_ with various substrates. The uptake rate of [^3^H] deoxy-D-glucose in oocytes expressing PvHT1_NK_ was significantly higher than in the control oocytes (**Figure 2**). However, other substrates, including [^3^H] arginine, [^3^H] glycine, [^14^C] α-ketoglutaric acid, [^14^C] lactate, and [^14^C] pyruvate showed no significant uptake activity (**Figure 2**).

**Figure 3:**
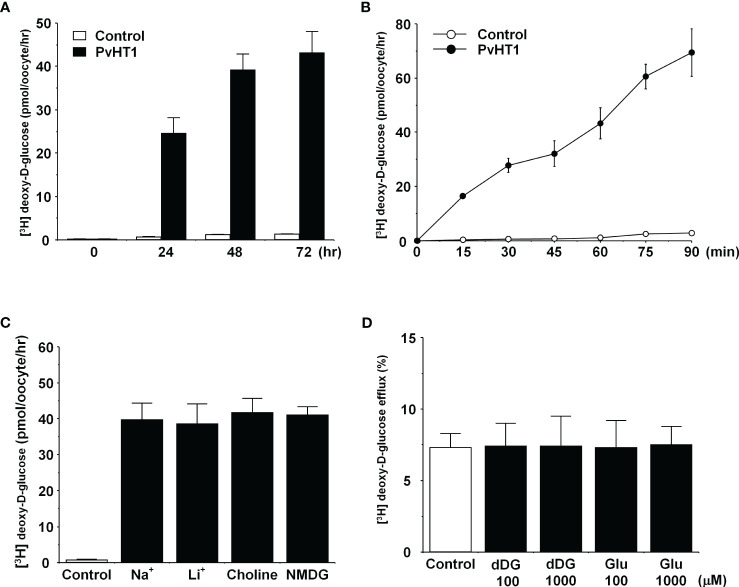
The transport properties of D-glucose via PvHT1_NK_. **(A)** The uptake of 300 nM [^3^H] deoxy-D-glucose in the PvHT1_NK_ expressing oocytes or water-injected (control) oocytes was measured at indicated PvHT1_NK_ expression times. **(B)** The uptake of 300 nM [^3^H] deoxy-D-glucose in control oocytes (open circle) and PvHT1**_NK_** expressing oocytes (closed circle) was measured by 15 min interval incubation time point up to 90 min. **(C)** Effect of extracellular cation on [^3^H] deoxy-D-glucose uptake in PvHT1**_NK_**-expressing oocytes. The uptake rate of [^3^H] deoxy-D-glucose (300 nM) was measured in the presence or absence of extracellular Na^+^. Extracellular Na^+^ was replaced with an equimolar concentration of lithium (LiCl), choline, and NMDG (N-methyl-d-glucosamine). **(D)** The lack of a trans-stimulatory effect of D-glucose on PvHT1**_NK_** mediated efflux of [^3^H] deoxy-D-glucose was observed. Oocytes expressed with PvHT1**_NK_** were incubated with 300 nM [^3^H] deoxy-D-glucose for 90 min and washed oocytes were transferred to the ND96 solution (control) or ND96 containing 100 uM or 1,000 uM unlabelled deoxy-D-glucose or Glucose. The efflux amount of D-glucose during 1 h was shown as the percentage of the preloaded amount. All results were represented by mean ± S.E. (*n* = 6-8).

The transport properties of [^3^H] deoxy-D-glucose via PvHT1_NK_ were confirmed through *trans*-uptake experiments. The *trans*-uptake activity of PvHT1_NK_ increased in an expression time (1 – 3 days)- and incubation time (15 min – 90 min)-dependent manner (**Figures 3A, B**). These results indicate that PvHT_NK_ both binds and translocates [^3^H] deoxy-D-glucose into the oocyte. To confirm sodium dependency, the sodium content in the uptake solution (ND96, containing 96 mM Na^+^ ions) was replaced with the same concentration of lithium ions, choline, and NMDG. The uptake of [^3^H] deoxy-D-glucose by PvHT_NK_ did not change after 1 hour, confirming its sodium-independent properties (**Figure 3C**). We also investigated the *trans*-stimulatory effect of PvHT1_NK_. The efflux of preloaded [^3^H] deoxy-D-glucose was not stimulated by radio-unlabelled deoxy-D-glucose and glucose (**Figure 3D**). These results reflect that PvHT1_NK_ is uniporter and highly selective for glucose.

**Figure 4:**
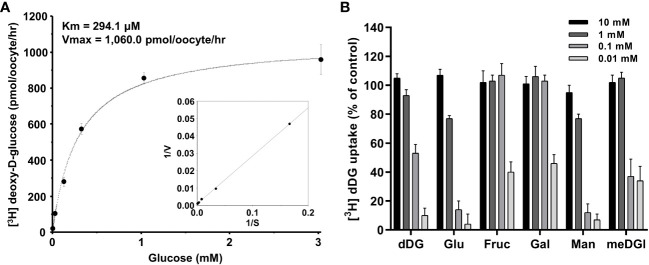
The saturation of PvHT1_NK_-mediated uptake of [^3^H]deoxy-D-glucose and active analogue on PvHT_NK_. **(A)** The uptake rates of [^3^H] deoxy-D-glucose by control (water-injected) or PvHT1**_NK_**-expressing oocytes for 1 h were measured at variable concentrations (mean ± S.E.; *n* = 6-8). Inset, Lineweaver-Burk analysis of concentration-dependent uptake of [^3^H]deoxy-D-glucose. V, velocity; S, the concentration of D-glucose. **(B)** Uptake assays were carried out with [^3^H] deoxy-D-glucose with various concentrations of sugar derivates. dDG, deoxy-D-glucose; Glu, Glucose; Fruc, Fructose; Gal, Galactose; Man, Mannose; meDG, methyl-D-Glucose.

The concentration-dependent uptake of [^3^H] deoxy-D-glucose mediated by PvHT1_NK_ exhibited saturable kinetics and followed the Michaelis-Menten equation. Nonlinear regression analysis yielded a *Km* value of 294.1 µM with a *Vmax* value of 1,060 pmol/oocyte/hr (**Figure 4A**). To investigate the selectivity of monosaccharide sugars for PvHT1_NK_, an inhibition study was conducted. Strong selectivity was observed with deoxy-D-glucose, glucose, and mannose, which inhibited [^3^H]deoxy-D-glucose uptake by 10 ± 5%, 4 ± 7%, and 7 ± 4%, respectively, at the highest concentration (**Figure 4B**). Additionally, Methyl-D-glucose showed moderate inhibitory activity at 1 mM. In contrast, fructose and galactose showed the lowest selectivity, inhibiting uptake by 40 ± 7% and 46 ± 6%, respectively, at 10 mM (**Figure 4B**).

### The glucose binding model of PvHT1_NK_

**Figure 5:**
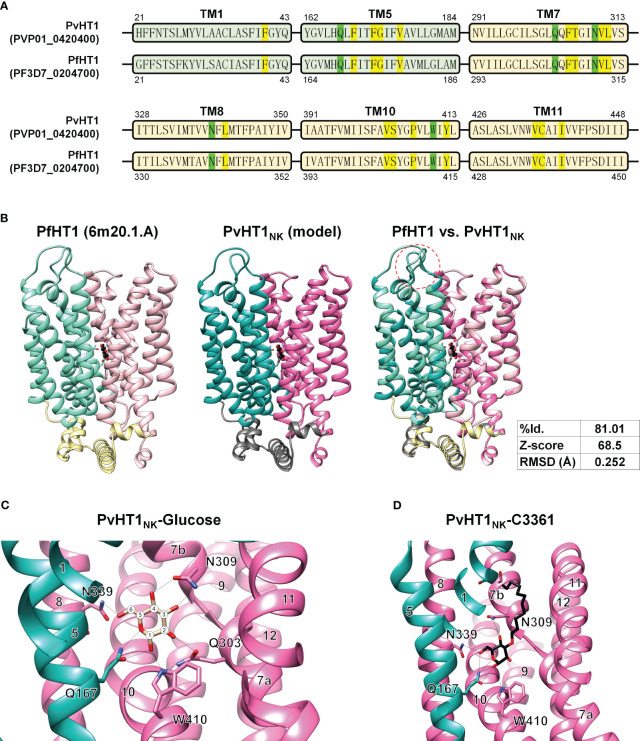
The glucose and C3361 binding mode of PvHT1_NK_. **(A)** The primary amino acid sequence comparison between PvHT1 and PfHT1 reveals that TM1 and TM5 localize in the NTD, with one critical amino acid (Q167, shown in green) responsible for glucose binding, along with five other amino acids involved in the glucose binding pocket (highlighted in yellow). In the CTD, which includes TM7, TM8, TM10, and TM11, there are four critical glucose-binding residues (Q303, N309, N339, and W410, shown in green), along with twelve amino acids forming the glucose-binding pocket (highlighted in yellow). These essential glucose-binding residues are well conserved. **(B)** The tertiary structure comparison between PfHT1 (PBD ID: 6m20.1A) and PvHT1_NK_ revealed several key findings. In the central panel, both PfHT1 (right panel) and PvHT1 _NK_ (central panel) exhibited an internal glucose binding mode located in the central part of the structure. Furthermore, when PfHT1 and PvHT1 _NK_ were superimposed, a high Z-score value of 68.5 and a significant RMSD score of 0.252 Å were obtained, indicating a substantial structural similarity. However, a notable difference in structure was observed in the loop connecting the extracellular region between TM1 and TM2 (left panel). **(C)** The coordination of D-glucose in PvHT1_NK_ is depicted in the structure, with the number of transmembrane (TM) segments and critical residues for glucose binding highlighted. The number of carbons is represented as numeric circles, and hydrogen bonds are illustrated as black lines. **(D)** The coordination of C3361 in PvHT1_NK_ is depicted in the structure, with the number of transmembrane (TM) segments and critical residues for C3361 binding highlighted. The hydrogen bonds are illustrated as black lines.

The putative glucose binding pocket was predicted by amino acid sequence alignment and the tertiary structure of PfHT1 (PDB ID: 6m20.1) as a homology model. The tertiary structure of PvHT1_NK_ revealed conserved amino acid residues across multiple transmembrane domains. The glucose binding pocket comprises five residues: F40, F169, F172, G173, and V176 in the NTD (**Figure 5A**). The CTD forms a glucose binding pocket composed of twelve residues: F305, T306, V310, L311, L341, V403, S404, P407, Y412, V435, C436, and I439 (**Figure 5A**). These amino acids have the same sequence as PfHT1, which contributes to a similar functional activity in glucose uptake by PvHT1_NK_. The structural comparison of tertiary conformations revealed a high degree of conservation between PvHT1_NK_ and PfHT1. In the structured domain, these two structures exhibited a substantial sequence identity of 81.01% (**Figure 5B**). Furthermore, the calculated Z-score of 68.5 significantly exceeded the threshold of 2, indicating the robustness of this structural similarity (**Figure 5B**). Additionally, the RMSD value was found to be 0.252 Å, which is less than 2.0 Å (**Figure 5B**). Therefore, the structures of PvHT1_NK_ and PfHT1 were well conserved and superimposed. The structural differences between PvHT1_NK_ and PfHT1 were revealed in the extracellular domain within the TM1 and TM2 connecting loop (**Figure 5B**).

Due to this superimposed tertiary structure, it is possible to estimate the critical glucose binding residues. The most essential residues for glucose binding are revealed to be Q167, Q303, N309, N339, and W410 (**Figure 5C**). Each hydroxyl group on glucose is coordinated through at least one hydrogen bond with these critical residues (**Figure 5C**). Similarly, the compound C3361 (also known as 3361), a glucose analog used for inhibiting PfHT1 glucose binding, exhibited similar binding properties in PvHT1_NK_ (**Figure 5D**).

## Discussion

The treatment of *Plasmodium vivax* has undergone minimal changes over nearly seven decades. Despite recommendations from the World Health Organization (WHO) to use artemisinin combination therapy (ACT), many endemic areas continue to use chloroquine (CQ) as the first-line treatment due to its cost-effectiveness. However, this cost-effective approach has led to the emergence and rapid spread of CQ-resistant vivax malaria (Price et al., 2014). As drug resistance continues to rise, there is a clear and urgent demand for novel antimalarial drugs development. To overcome these challenges, researchers are actively exploring novel molecular targets and mechanisms of action (Flannery et al., 2013).

*Plasmodium* species primarily rely on glucose as an essential nutrient in all stages of their lifecycle (Salcedo-Sora et al., 2014). The HT1 function was confirmed for glucose uptake, and the lack of glucose affect *P. falciparum* growth (Joet et al., 2003a; Saliba et al., 2004; Blume et al., 2011). Subsequently, the strategy of blocking glucose uptake via HT1 has shown success in reducing the replication of parasites in the blood stage (Joet et al., 2003a). This success was demonstrated using compound 3361 (C3361), a long-chain O-3 glucose derivative that competes at the binding site of PfHT1 (Joet et al., 2003a; Joet et al., 2003b). The host cell utilizes GLUT1 for glucose uptake, and this transporter shares a relatively low sequence similarity of 22.6%~23.4% with HT1 in various human malaria *Plasmodium* species (**Supplementary Figure 1**) (Joet et al., 2003a; Joet et al., 2004). Additionally, the binding pocket residues show significant differences between PfHT1 and hGLUT1, with PfHT1 being highly hydrophobic and hGLUT1 being hydrophilic (Huang et al., 2021). Thus, it induced approximately 40-fold different selectivity for C3361, as shown by the IC_50_ values of 33.1 ± 2.0 μM for PfHT1 and 1.3 mM for hGLUT (Jiang et al., 2020). Thus, HT1 becomes an attractive target for designing drugs with a novel mechanism of action (Joet and Krishna, 2004). The primary amino acid sequences of these transporters in human malaria *Plasmodium* species show at least 76.4% identity between PfHT1 and PkHT1 (**Supplementary Figure 1**). Furthermore, when comparing sequences within *P. vivax* at the intra-species level, identical residues were found in the glucose binding pocket. Natural variations occur exclusively in extracellular domain residues. These bioinformatic results support the essential role of conserved residues in glucose uptake and the binding mode of inhibitor C3361 across *Plasmodium* species (Joet et al., 2002; Joet et al., 2004).

The uptake properties of PvHT1_NK_ demonstrated a high affinity of D-glucose with sodium-independency. Typically, high-affinity glucose transporters show sodium independency in various organisms (Woodrow et al., 1999; Joet et al., 2002; Vemula et al., 2009). Additionally, PvHT1_NK_ was identified as a uniporter in the efflux experiment. Sugar selectivity was observed, with a high affinity for glucose and mannose, moderate affinity for methyl-D-glucose, and low affinity for fructose and galactose. These properties are similar to PfHT1 (Woodrow et al., 1999; Woodrow et al., 2000). Therefore, C3361 has the potential to replace glucose, thereby interrupting glucose uptake via PvHT1_NK_ (Joet et al., 2004). It has been demonstrated that a single residue in predicted helix 5, specifically the mutation of position Q167 to asparagine (N), determines fructose specificity but does not affect glucose uptake in *P. falciparum* (Huang et al., 2021). Furthermore, this residue mutation led to decreased susceptibility to inhibition by C3361 in PvHT1 (Woodrow et al., 2000; Joet et al., 2003a). Based on the structure prediction analysis for PvHT1_NK_, Q167 strongly interacts with the C3361 by hydrogen bond that possibly affects this interaction strength. In the sequence comparison across inter- and intra-species levels, no point mutations were detected in Q167, which is consistently associated with low affinity for fructose uptake. Previous report also confirmed that the inhibition of *P. vivax* schizont maturation required C3361 concentrations within a similar IC_50_ range (9–58 μM) to those needed for inhibiting the maturation of *P. falciparum* (IC_50_ range 12–75 μM) (Joet et al., 2003b; Joet et al., 2004). However, a significant drawback of C3361 is its cytotoxicity at concentrations over 50 μM in HEK293/17 and HepG2 cell lines (Jiang et al., 2020). Therefore, recent efforts have focused on C3361 derivatives for antiparasitic activities, and HTI-1 has been suggested as a promising drug development candidate with a lower IC_50_ and higher cytotoxicity (CC_50_) (Jiang et al., 2020). In conclusion, the development of HT1 inhibitors as novel antimalarials has the potential to be effective against both *P. falciparum* and *P. vivax*.

Biologically, the difference between PvHT1 and PfHT1 has been observed in their expression levels during blood stage maturation. In *P. falciparum*, it has been confirmed that mRNA levels peak sharply 8 hours after the invasion of the host cell, reflecting a dramatic increase in glucose consumption required for proper maturation (Woodrow et al., 1999). In the case of PvHT1, delayed peaks were observed 13 hours after invasion (Bozdech et al., 2008). Compared with host cell preference, *P. vivax* primarily invades young reticulocytes. Based on host cell maturation, the expression level of GLUT1 also gradually increases (Montel-Hagen et al., 2008). Thus, in the case of *P. vivax* at the early infection stage, there is a lower concentration of glucose in the host cell. One hypothesis suggests that to overcome the lack of glucose, PvHT1 should have a higher affinity (Km=294 μM) for glucose uptake than *P. falciparum* (Km=1,000 μM), allowing it to use less energy more efficiently (Woodrow et al., 2000; Joet et al., 2003a).

In summary, the present study characterizes the structural and functional aspects of PvHT1_NK_. The identification of conserved glucose-binding residues and the cross-species activity of certain inhibitors offer promising prospects for future antimalarial drug development. Further research is required to explore the precise mechanisms of glucose uptake in various *Plasmodium* species for the development of novel therapeutics.

## Data availability statement

The raw data supporting the conclusions of this article will be made available by the authors, without undue reservation.

## Ethics statement

The studies involving humans were approved by the Institutional Review Board (IRB) of Inha University. The studies were conducted in accordance with the local legislation and institutional requirements. The human samples used in this study were acquired from a by- product of routine care or industry. Written informed consent for participation was not required from the participants or the participants’ legal guardians/next of kin in accordance with the national legislation and institutional requirements.

## Author contributions

JW: Formal analysis, Investigation, Validation, Visualization, Writing – original draft. ME: Data curation, Investigation, Writing – review & editing, Validation. SC: Conceptualization, Data curation, Funding acquisition, Project administration, Supervision, Validation, Visualization, Writing – review & editing. J-HH: Conceptualization, Data curation, Formal analysis, Funding acquisition, Investigation, Supervision, Validation, Visualization, Writing – original draft, Writing – review & editing.

## Supplementary material

Supplementary Figure 1 Percentage identity shared among amino acid sequences of hexose transporters shown in the alignment. The relationship is shown between the HT1 of human malaria parasite; *P. malariae* (Pm), *P. ovale* curtisi (Poc), *P. ovale* wallikeri (Pow), *P. knowlesi* (Pk) and hGLUT1 sequences as shown in the alignment (values are in percentages).

Supplementary Figure 2 Sequence alignment of PvHT1 within intra-species level. The sequence conservation ratio is represented by a gradient bar from red to blue. Essential residues for the glucose binding pocket are highlighted in yellow within the amino acid positions, while critical residues for glucose binding are shown in green highlights. Residues that match with North Korean strain exactly hide as “.”.

Supplementary Figure 3 Sequence alignment of PvHT1 with homologs in human malaria *Plasmodium* species. The sequence conservation ratio is represented by a gradient bar from red to blue. The N-terminal transmembrane domain (NTD) with a helical structure is depicted in deep green, the intracellular region with four alpha-helices (ICH) is shown in grey, and the C-terminal transmembrane domain (CTD) is depicted in deep pink. Essential residues for the glucose binding pocket are highlighted in yellow within the amino acid positions, while critical residues for glucose binding are shown in green highlights.
