# Supplementary figures and images for: Functional characterization of Plasmodium vivax hexose transporter 1
Source: Front Cell Infect Microbiol. 2024 Jan 12;13:1321240. doi: 10.3389/fcimb.2023.1321240 (PMC10811246; doi:10.3389/fcimb.2023.1321240)

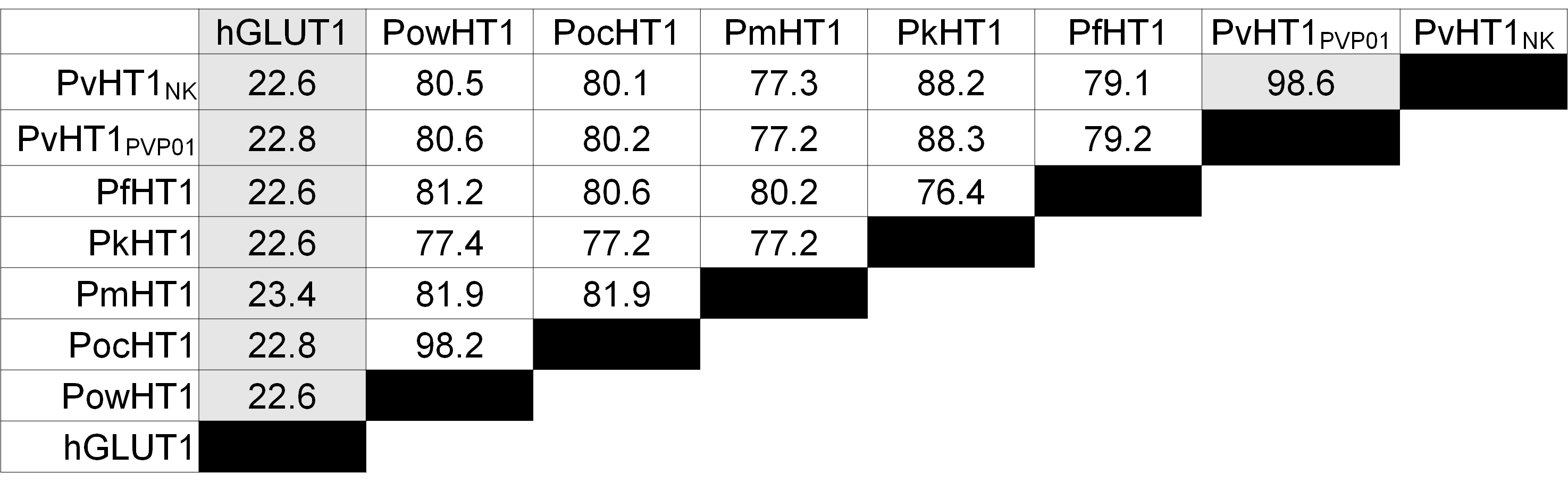

Supplement: Supplementary Figure 1 — Percentage identity shared among amino acid sequences of hexose transporters shown in the alignment. The relationship is shown between the HT1 of human malaria parasite; P. malariae (Pm), P. ovale curtisi (Poc), P. ovale wallikeri (Pow), P. knowlesi (Pk) and hGLUT1 sequences as shown in the alignment (values are in percentages). [file Image_1.tif]

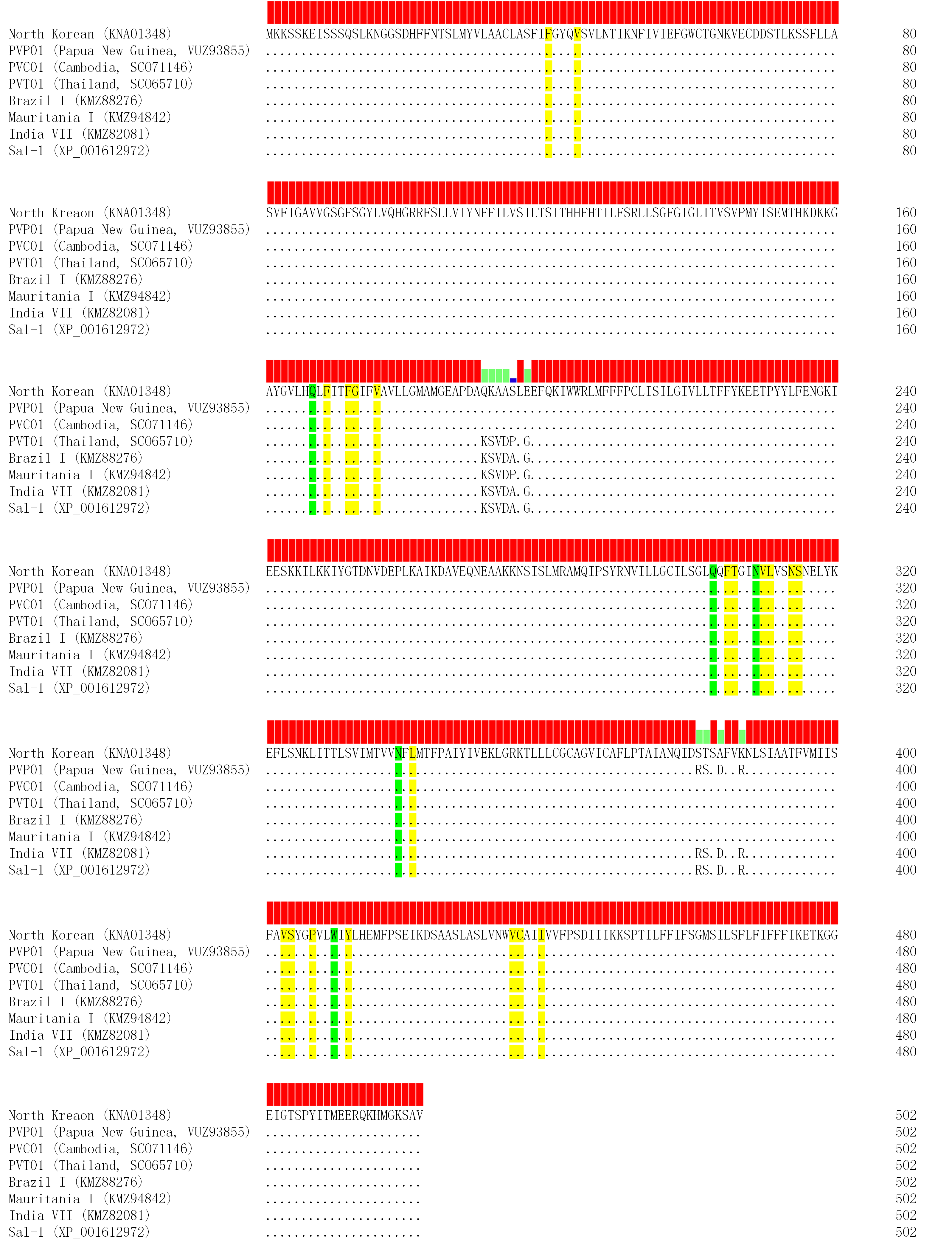

Supplement: Supplementary Figure 2 — Sequence alignment of PvHT1 within intra-species level. The sequence conservation ratio is represented by a gradient bar from red to blue. Essential residues for the glucose binding pocket are highlighted in yellow within the amino acid positions, while critical residues for glucose binding are shown in green highlights. Residues that match with North Korean strain exactly hide as “.”. [file Image_2.tif]

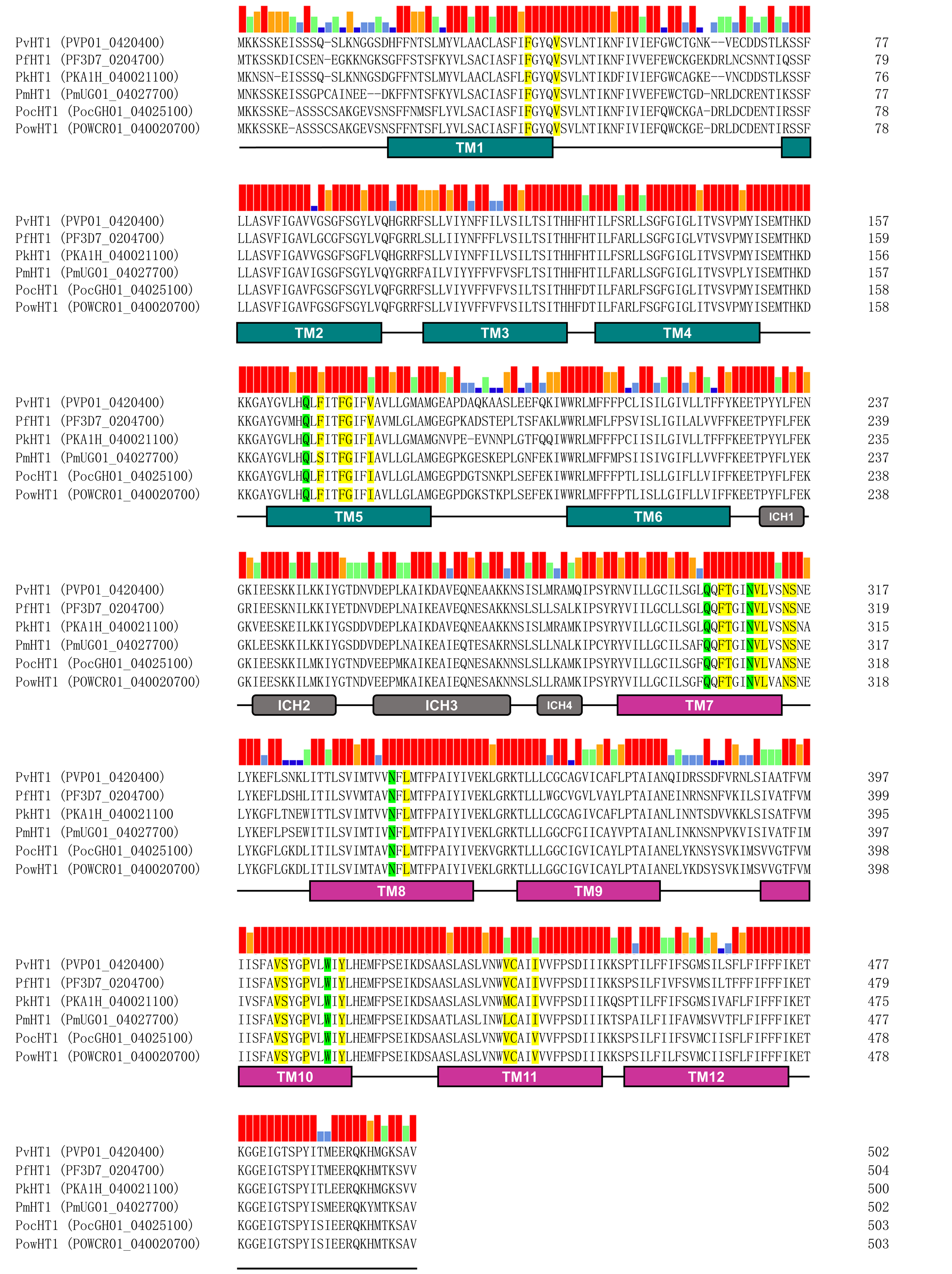

Supplement: Supplementary Figure 3 — Sequence alignment of PvHT1 with homologs in human malaria Plasmodium species. The sequence conservation ratio is represented by a gradient bar from red to blue. The N-terminal transmembrane domain (NTD) with a helical structure is depicted in deep green, the intracellular region with four alpha-helices (ICH) is shown in grey, and the C-terminal transmembrane domain (CTD) is depicted in deep pink. Essential residues for the glucose binding pocket are highlighted in yellow within the amino acid positions, while critical residues for glucose binding are shown in green highlights. [file Image_3.tif]
